# Untargeted Metabolomics-Based Screening Method for Inborn Errors of Metabolism using Semi-Automatic Sample Preparation with an UHPLC- Orbitrap-MS Platform

**DOI:** 10.3390/metabo9120289

**Published:** 2019-11-26

**Authors:** Ramon Bonte, Michiel Bongaerts, Serwet Demirdas, Janneke G. Langendonk, Hidde H. Huidekoper, Monique Williams, Willem Onkenhout, Edwin H. Jacobs, Henk J. Blom, George J. G. Ruijter

**Affiliations:** 1 Center for Lysosomal and Metabolic Diseases, Department of Clinical Genetics, Erasmus University Medical Center, Dr. Molewaterplein 40, 3015 GD Rotterdam, The Netherlands; m.bongaerts@erasmusmc.nl (M.B.); s.demirdas@erasmusmc.nl (S.D.); w.onkenhout@erasmusmc.nl (W.O.); e.jacobs@erasmusmc.nl (E.H.J.); h.j.blom@erasmusmc.nl (H.J.B.); 2 Center for Lysosomal and Metabolic Diseases, Department of Internal Medicine, Erasmus University Medical Center, Dr. Molewaterplein 40, 3015 GD Rotterdam, The Netherlands; j.langendonk@erasmusmc.nl; 3 Center for Lysosomal and Metabolic Diseases, Department of Pediatrics, Erasmus University Medical Center, Dr. Molewaterplein 40, 3015 GD Rotterdam, The Netherlands; h.huidekoper@erasmusmc.nl (H.H.H.); m.williams@erasmusmc.nl (M.W.)

**Keywords:** metabolomics, inborn errors of metabolism, LC-MS, HRAM-MS, Orbitrap, PFPP, IEM, organic aciduria, urea cycle defects, PKU

## Abstract

Routine diagnostic screening of inborn errors of metabolism (IEM) is currently performed by different targeted analyses of known biomarkers. This approach is time-consuming, targets a limited number of biomarkers and will not identify new biomarkers. Untargeted metabolomics generates a global metabolic phenotype and has the potential to overcome these issues. We describe a novel, single platform, untargeted metabolomics method for screening IEM, combining semi-automatic sample preparation with pentafluorophenylpropyl phase (PFPP)-based UHPLC- Orbitrap-MS. We evaluated analytical performance and diagnostic capability of the method by analysing plasma samples of 260 controls and 53 patients with 33 distinct IEM. Analytical reproducibility was excellent, with peak area variation coefficients below 20% for the majority of the metabolites. We illustrate that PFPP-based chromatography enhances identification of isomeric compounds. Ranked z-score plots of metabolites annotated in IEM samples were reviewed by two laboratory specialists experienced in biochemical genetics, resulting in the correct diagnosis in 90% of cases. Thus, our untargeted metabolomics platform is robust and differentiates metabolite patterns of different IEMs from those of controls. We envision that the current approach to diagnose IEM, using numerous tests, will eventually be replaced by untargeted metabolomics methods, which also have the potential to discover novel biomarkers and assist in interpretation of genetic data.

## 1. Introduction

Routine diagnostic testing for inborn errors of metabolism (IEM) in clinically selected individuals is currently performed by targeted analysis of known biomarkers [1]. In most laboratories, specific groups of metabolites, such as organic acids, amino acids and acylcarnitines, are analyzed by dedicated platforms (e.g., GC-MS, HPLC-DAD/UV and LC-MS/MS). These dedicated assays require multiple preparations of different types of samples, such as urine, plasma or cerebrospinal fluid. This diagnostic process is laborious, time-consuming, expensive, and delays diagnosis and, consequently, specific treatment. Targeted assays including multiple metabolite groups have been developed to address these issues [2,3]. Targeted assays are optimized for the physicochemical properties of the biomarkers and the matrices of the samples, which, in general, result in reliable (semi-) quantitative analyses. As a consequence, a dedicated assay can never provide a comprehensive overview of the patient’s metabolome, as (*i*) a limited number of dedicated tests will not determine those known biomarkers that require alternative platforms and (*ii*) targeted assays fail to identify novel biomarkers and new IEM.

Diagnostic use of untargeted metabolomics has only very recently become feasible, due to recent advances in UHPLC and high-resolution accurate mass spectrometry (HRAM)-MS. Especially the ultra-high resolution of Orbitrap-MS enables the accurate detection of molecular ions (<1 ppm mass deviations) and the resolution of their fine isotope patterns, allowing unsurpassed specificity. As a consequence, untargeted metabolomics have become a promising technique, with the potential to overcome the disadvantages of targeted assays mentioned above [4,5,6,7]. It offers the determination of hundreds to thousands of small molecules in a single experiment, allowing the detection of numerous molecules—substrates, intermediates and final products—in metabolic pathways.

Only few untargeted metabolomics-based methods to screen for IEM have been published. The first published method applied GC-MS and LC-high resolution MS run in parallel [8]. The second [9] and the third [10] methods used LC-QTOF/MS. The fourth metabolomics assay is based on Orbitrap-MS without using chromatographic separation [11]. Although applications of untargeted metabolomics in IEM screening are emerging, they are still in an exploratory phase and various challenges need to be addressed, such as high-throughput sample preparation, identification and separation of isobaric and isomeric molecular species, and data-processing.

Here, we describe a powerful novel single platform untargeted metabolomics method that we developed for screening of IEM. We illustrate that pentafluorophenylpropyl phase-based UHPLC coupled to ultra-high mass resolution Orbitrap-MS enhances separation of isobaric and isomeric metabolites. We describe the reproducibility of test results and evaluate the diagnostic capacity of the method by analyzing plasma samples of 53 patients with 33 distinct IEM in different metabolic pathways.

## 2. Results

### 2.1. Analytical Performance

Plasma samples were analyzed in the positive as well as in the negative ionization mode in eight independent batches, including a total of 260 control samples and 53 IEM patient samples. More than 2 × 10^6^ peaks were detected in the raw data of each sample. Each batch was separately processed, resulting in the detection of more than 17,256 (range: 17,256–26,775) compound ions (i.e., an analytical feature with a specific *m*/*z* ratio, intensity, retention time and its corresponding isotope pattern signals). To explore potential IEM disease markers, the >17,250 compound ions detected in each plasma sample were matched against 339 clinically relevant biomarkers included in our in-house database. On average, 178 metabolite annotations were made in each of the 16 experiments (range 140–244). Mass accuracy of these annotations was always within 3 ppm, while 78.6% were within 1 ppm.

### 2.2. Retention Time Stability

To investigate the chromatographic stability of the LC method, we monitored within-batch and between-batch retention time (RT) variation of 17 stable isotope-labeled standards, which were added to the samples. Within batch RT variation was never higher than 1.12% for the positive ion mode and 0.37% for the negative ion mode, while the median CV of within batch variation was 0.22% (all plasma samples and QC samples, N = 29–166). Median and range data for all internal and external standards of all batches are shown in supplementary materials S1. Between batch RT variation was within 2% (all plasma samples and QC samples), except for [D_10_]-isoleucine (3.8%, N = 1011), [D_2_]-uridine (4.7%, N = 1011), [D_3_]-methylmalonic acid (5.3%, N = 504), and [D_4_]-tyrosine (5.4%, N = 1011), which were all eluting between 1.70–2.80 min. This larger variation probably relates to the use of two analytical columns (although manufactured with core-shell particles from the same batch) during the experiments, since RT variation between different batches analyzed on one column was always within 2%. Between-batch RT variation data for all internal and external standards are shown in supplementary materials S2.

### 2.3. Variation in Peak Area

Median within-batch CV values of peak areas of internal and external standards in all plasma and QC samples were 7–19% (range 4–30%, N = 29–83). Relatively large variations were observed for [D_3_]-tetradecanoylcarnitine and [D_3_]-hexadecanoylcarnitine, with median CVs over the eight batches of 19.0% and 19.4%, respectively (range 13.1–30.3%, N = 29–83). Within-batch median CVs and ranges for all internal and external standards in both ion modes are shown in supplementary materials S3. Median between-batch variation of all standards in all plasma and QC samples was 27% (range 17–68%, N = 504–1011). Between-batch median CVs of all internal and external standards in all samples are shown in supplementary materials S4.

**Figure 1:**
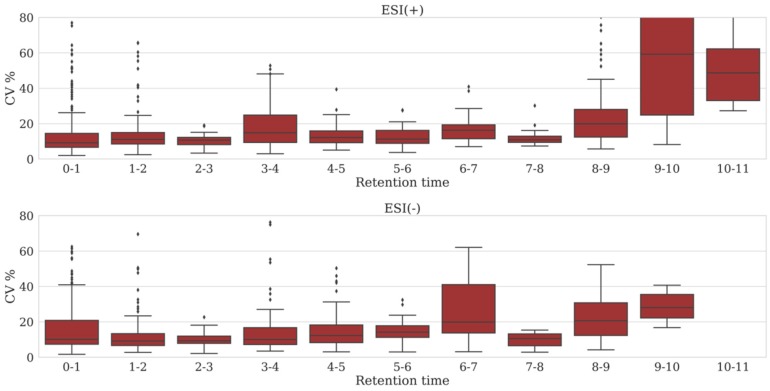
Variation in peak area across the chromatographic run. Box-plots of within batch CVs in peak areas from all eight batches are shown containing all metabolites annotated in the QC samples, binned by retention time (bin width: 1 min); see text. Top panel: positive ion mode, bottom panel: negative ion mode.

To obtain more information regarding the reproducibility of peak areas across a chromatographic run, we monitored peak areas of metabolites that were consistently annotated in all analyses of the QC sample across the eight batches (supplementary materials S8). Within-batch CVs were determined per metabolite and binned by retention time (bin width: 1 min), covering the whole chromatogram for all eight batches. For each batch, this resulted in 11 bins and 10 bins containing a total of 119 and 87 metabolites, for the positive and negative ion mode, respectively. First, we reviewed each bin in the eight batches separately, i.e., 88 bins for the positive ion mode and 80 bins for the negative ion mode. Median CV of variation in peak area in the positive ion mode was <30% for 73/88 bins (83%) and 66/88 bins (75%) had a median CV <20%. In the negative ion mode, 73/80 bins (91%) had a median CV <30%, while in 62/80 bins (78%) median CV was <20%. The largest variation was observed near the end of the chromatogram in bins 8–9, 9–10 and 10–11 min (supplementary materials S5). Next, we reviewed median CVs across all batches for each bin (see Figure 1). A constant low variation in the peak areas measured in a bin across batches suggests a stable LC-MS method. Only bins 8–9, 9–10 and 10–11 min in the positive ion mode showed a median CV >20%, while in the negative ion mode a median variation >20% was observed in bins 6–7, 8–9 and 9–10 min (Figure 1). Overall, 182 of the 206 (88%) metabolites annotated had a median CV <20%.

### 2.4. Separation of Isobaric and Isomeric Species

Correct annotation of features is crucial in the diagnostics of IEM, because misidentification or lack of identification can result into false positive or false negative results. In this respect, it is challenging to make the correct annotation of isobaric and isomeric species. Using our Orbitrap-MS, a resolution of 140,000 is feasible while maintaining adequate data sampling frequency (1.8 scans/s). To determine the ability to separate isobaric ions by using only mass spectrometry, we identified 1608 combinations of isobaric ions derived from metabolites present in our in-house database, which required a resolution >40,000 for separation. In the ESI(+)-mode 1032 out of 1608 combinations (64%) could be baseline-separated by acquiring the data at a resolution of 140,000. This included, for example, serine[K^+^] and cysteine[Na^+^], that co-elute in our LC-method. In the ESI(-)-mode, 1010 out of 1608 combinations (63%) could be resolved by acquiring the data at a resolution of 140,000 (detailed data available on request).

**Figure 2:**
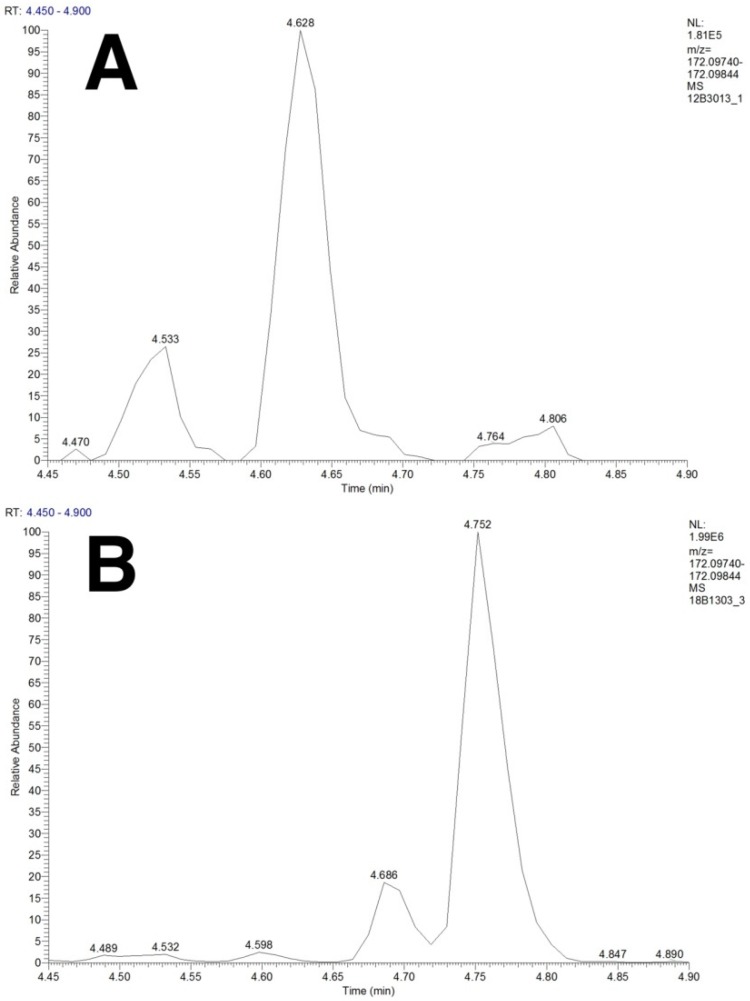
Separation of isomeric compounds using pentafluorophenylpropyl-based UHPLC. Panel (**A**) XIC (mass tolerance: 1 ppm, ionization: ESI(-)) of 172.09792 *m*/*z* in an aminoacylase I deficiency sample showing peaks of *N*-acetylisoleucine (RT: 4.533 min) and *N*-acetylleucine (RT: 4.628 min). Panel (**B**): XIC (mass tolerance: 1 ppm, ionization: ESI(-)) of 172.09792 *m*/*z* in a medium chain acyl-CoA dehydrogenase deficiency sample showing peaks of *N*-acetylleucine (RT: 4.598 min), isohexanoylglycine (RT: 4.686 min) and hexanoylglycine (RT: 4.752 min).

To achieve correct annotation of isomeric compounds, chromatographic separation is required. We selected pentafluorophenylpropyl (PFPP) phase-based chromatography, because PFPP, as a stationary phase, shows better retention for small polar compounds, such as organic acids and amino acids, compared to traditional C18-based chromatography and is more stable in terms of retention time variation and required stabilization times, compared to HILIC [12,13,14,15,16]. An example of compounds that are separated by our LC-method is the isomeric group *N*-acetylisoleucine, *N*-acetylleucine, isohexanoylglycine and hexanoylglycine (C_8_H_15_NO_3_). Separation of these isomers is required to distinguish aminoacylase I deficiency from medium chain acyl-CoA dehydrogenase deficiency. Figure 2 clearly shows separation of *N*-acetylisoleucine and *N*-acetylleucine in a plasma sample of a patient with aminoacylase I deficiency, while hexanoylglycine and isohexanoylglycine peaks are present at different retention times in a plasma sample of a patient with medium chain acyl-CoA dehydrogenase deficiency. Other examples of compounds that can be separated by our LC method using PFPP chromatography are the isomeric pairs isoleucine/leucine and betaine/valine (see supplementary material S6), as well as 2-hydroxybutyric acid/3-hydroxybutyric acid/4-hydroxybutyric acid (C_4_H_8_O_3_), glutaric acid/ethylmalonic acid/methylsuccinic acid (C_5_H_8_O_4_) and tiglylglycine/3-methylcrotonylglycine (C_7_H_11_NO_3_) (data not shown).

### 2.5. Data Analysis and IEM Detection

**Figure 3:**
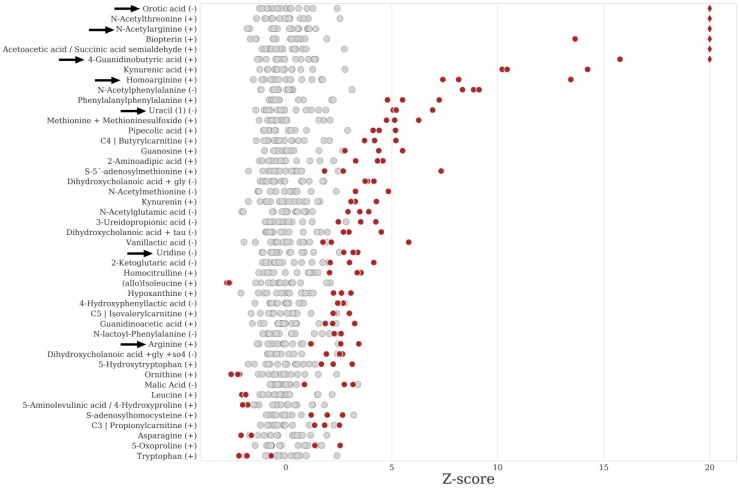
Metabolomics test result of a hyperargininemia sample. The top 45 of ranked z-scores are depicted with arrows highlighting metabolites relevant to the diagnosis. Red diamonds: average z-score of the patient triplicate >20, red dots: z-scores of the patient triplicate, grey dots: reference values (*n* = 15).

We have studied plasma samples of 53 patients covering 33 distinct IEM to evaluate our UHPLC-Orbitrap-MS platform and data processing pipeline. We investigated the ability to (1) detect metabolites relevant to IEM and (2) to produce metabolite signatures that can be interpreted to assign a diagnosis. Z-scores of annotated metabolites were calculated using 15 age - and sex-matched control samples originating from the same batch as the patient samples (Table 1). Ranked z-score plots (metabolite with highest z-score at the top), representing metabolic signatures of the 53 patient samples analyzed, were reviewed in a blinded fashion and independently by two laboratory specialists experienced in clinical biochemical genetics to assign the most likely diagnosis. The z-score plot obtained for a hyperargininemia sample is shown in Figure 3 as an example. In 95 of the 106 reviews (90%), the correct diagnosis was achieved. Two diagnoses remained undetected: alkaptonuria and mevalonic aciduria. The known diagnostic biomarkers for these diseases, homogentisic acid and mevalonic acid, were found, but the levels were not significantly different compared to the controls (Table 1). Surprisingly, the urinary homogentisic acid level of the alkaptonuria patient sampled on the same day was strongly elevated (3074 mmol/mol creatinine; reference values <5). Diagnoses were incorrect in some of the cases reviewed for the following IEM: carbamylphosphate synthase I deficiency (two out of four reviews), ornithine transcarbamylase deficiency (1/4), homocystinuria (1/6), tyrosinemia type I (1/4), carnitine transporter deficiency (1/2) and 2-methyl-3-hydroxybutyryl-CoA dehydrogenase deficiency (1/2).

To highlight the unique features of untargeted metabolomics, which may result in the discovery of new biomarkers for diagnosis and therapy monitoring, we present a case of hyperargininemia. In the traditional targeted diagnostic process, hyperargininemia would be diagnosed if plasma levels of arginine are strongly elevated together with elevated plasma levels of glutamine and citrulline and high levels of urinary orotic acid [18]. We performed untargeted metabolomics on a plasma sample of a hyperargininemia patient treated by dietary protein restriction. As expected, arginine was only marginally increased, due to treatment, with a z-score of 2.4 and *p*-value > 0.05 (Figure 3, Table 1). Despite the treatment, we detected several elevated features, which were putatively annotated as 2-oxoarginine, *N*-acetylarginine, argininic acid, homoarginine and 4-guanidinobutyric acid by the Human Metabolite Database (HMDB). These metabolites have been reported previously in hyperargininemia patients [19,20,21,22]. Inclusion of these biomarkers in the panel of IEM metabolites resulted in high z-scores for *N*-acetylarginine, homoarginine and 4-guanidinobutyric acid in addition to orotic acid, a well-established biomarker of several urea cycle defects (Figure 3, Table 1). This example shows the strength of untargeted metabolomics analyses. The additional three biomarkers help to appoint the diagnosis hyperargininemia in this sample. Argininic acid and 2-oxoarginine remained undetected during the automated raw data analysis, but manual review of the raw data revealed elevated levels compared to controls. Argininic acid was not peak-picked in either ion mode by Progenesis QI, while 2-oxoarginine was not peak-picked in the negative ion mode and its peak incorrectly deconvoluted in the positive ion mode (the [M+H] adduct of 2-oxoarginine was annotated as the [M-H_2_O+H] adduct of 4-hydroxycitrulline).

## 3. Discussion

Mass spectrometry analytics has matured in recent decades. As a result, metabolomic studies have grown more precise and comprehensive, now allowing the identification of hundreds to thousands of unique metabolites in the analysis of a single biological sample [23]. The current study concerns a novel platform for untargeted metabolomics in diagnosing IEM based on semi-automatic sample preparation combined with pentafluorophenylpropyl phase-based (Kinetex F5) UHPLC coupled to Orbitrap-MS. The individual capabilities of each of these two analytical techniques will work synergistically and enable analysis of low-abundance components in complex samples and separation of isobaric and isomeric species. As an example, we showed the clear separation of the isomers isohexanoylglycine and hexanoylglycine and the isomers *N*-acetylleucine and *N*-acetylisoleucine (Figure 2). To minimize the chance of false positive and false negative results we used the Q Exactive Plus at a resolution of 140,000 (full half-maximum width at 200 *m*/*z*). The exceptional specificity of a resolution of 140,000 is of great importance. As we showed, 64% of the isobaric pairs that need a resolution higher than 40,000 will be separated at a resolution 140,000. Furthermore, the within batch quality control data show that, even with this ultra-high resolution, peak areas can be determined accurately. Last but not least, we used a semi-automatic sample preparation procedure. To the best of our knowledge, fully or semi-automated sample preparation procedures have not been used in metabolomics studies to diagnose IEM. Other methods have used manual protein precipitation and centrifugation [8,9,10,11]. Semi-automated sample processing potentially increases laboratory efficiency, reduces user errors and increases reproducibility. It must be noted that we did not investigate performance of semi-automated sample preparation in comparison to manual sample processing, but, from a practical point of view, automation did facilitate integration of sampling handling into the lab information system. An additional advantage of the 96-well Phree filter plates that we employed, is selective binding of phospholipids, which, if not removed, may cause ion suppression [24].

We have evaluated our method by testing 53 patient samples corresponding to 33 different IEM and achieved a correct diagnosis in 90% of the cases. Disorders from the following disease groups were included: aminoacidopathies, urea cycle disorders, organic acidurias, fatty acid oxidation defects, purine and pyrimidine disorders and peroxisomal disorders. For an additional IEM disease group—lysosomal storage disorders—the utility of clinical testing using metabolomics has not been not been reported before. We demonstrate that our metabolomics platform is able to detect mannosyl-β1,4-*N*-acetylglucosamine (GlcNAc-Man), the current biomarker for β-mannosidase deficiency. Preliminary experiments on oligosaccharidoses showed that biomarkers for aspartylglucosaminuria and α-mannosidosis were also easily detectable (data not shown).

A limitation of our platform was encountered in the raw data processing steps using Progenesis QI. Using a mass resolution of 140,000 allows the determination of the fine isotope pattern (N, O and S atoms) of molecules with *m*/z < 500, which helps to identify biomarkers by restricting the number of possible annotations. Unfortunately, Progenesis QI was unable to extract the fine isotope patterns, which were indeed correctly recorded by the orbitrap MS. Consequently, we could not use the fine isotope patterns to determine isotope similarity scores. For the annotated compounds, the isotope similarity score was always >85%, which we considered acceptable. Another drawback of Progenesis QI is that integration of picked peaks cannot be edited, e.g., to split isobaric compounds, which the algorithm integrated as a single peak, or to manually add peaks, which were missed by the peak detection algorithm. This resulted in missed annotations and the inability to report some isomers, which were, in fact, present in the raw data. Better procedures for peak-picking and deconvolution of adducts and isotope signals are required to optimize metabolite identification (see below).

Applications of untargeted metabolomics platforms to clinical testing in the field of IEM are scarce. Those platforms that used plasma samples include the approach reported by Miller et al., using GC-MS and LC-high resolution MS run in parallel [8], an LC-QTOF/MS method described by Coene et al. [9], and a direct infusion-Orbitrap-MS method reported by Haijes et al. [11]. Comparison of method performances is complicated, since many variations exist between the different approaches. Retention time stability observed on our PFPP column was ≤ 1.1% within batch and <2% between batches, which is very similar to the values (<1% within run and <2% between run) reported by Coene et al. [9] for the commonly used C18 UHPLC and indicates that very stable chromatography can be achieved on PFPP-based columns. Since the aforementioned metabolomics approaches rely on relative perturbations in metabolite levels, peak area detection must be reproducible across different samples. For the 17 stable isotope-labeled standards added to the samples, the within-batch median peak area CVs were 7–19% (data from all plasma samples included in a batch). These values compare well to the median CV values determined in a similar manner by Haijes et al. [11]: 16–21%. Coene et al. [9] reported a median CV of <20% for approximately 20 metabolites in their QC samples. We found that, in our method, the median within-batch CV values of peak areas of metabolites detected in the QC sample are well below 20% across the chromatogram (Figure 3). Only in the last two minutes of the chromatogram peak area CV values were larger, which may be explained by the fact that late-eluting compounds are apolar metabolites, e.g., long chain acylcarnitines, which are present at very low concentrations (<0.1 µmol/L) in the QC sample with limited solubility in the final diluent (water:methanol 95:5% + 0.5% *v*/*v* formic acid). The resulting fluctuations in metabolite recovery may be a major cause of the relatively high variation observed. Since these IEM screening methods investigate metabolite abnormalities within-batch, variation in peak areas between different experimental batches is less of an issue. Still, a stable platform is desirable and between-batch variation in peak areas should be monitored. Median between-batch variation of all standards in all samples was acceptable (27%).

A challenge in the use of untargeted metabolomics platforms to screen for IEM is the lack of adequate determination of some clinically relevant metabolites. Using our platform, homogentisic acid and mevalonic acid levels were not increased in an alkaptonuria sample and a hyper-IgD syndrome sample, respectively, and this resulted in failure to establish the diagnoses in these two cases. Similarly, a normal glutamine level was observed in one CPS I sample, which impeded correct diagnosis. Several other metabolites were not annotated or had normal values (Table 1), e.g., orotic acid in two OTC samples, but in these cases the correct diagnosis could still be established, because other metabolites had abnormal levels. Similar findings have been reported by other researchers. The platform described by Miller et al. [8] correctly diagnosed 20 out of the 21 IEM tested. Their method did not identify methylmalonic acid, tetradecenoylcarnitine (C14:1), and guanidinoacetic acid, but only in the latter case the diagnosis of guanidinoacetate methyltransferase (GAMT) deficiency was missed. Coene et al. [9] correctly identified 42 out of 46 diagnoses and could not diagnose argininosuccinate lyase deficiency, dimethylglycine dehydrogenase deficiency and GAMT deficiency, because abnormal values of argininosuccinic acid, dimethylglycine and guanidinoacetic acid were not annotated. A possible explanation for the inability to detect argininosuccinic acid was the lack of retention by C18 chromatography [9]. In our method, using PFPP-based chromatography, argininosuccinic acid was retained and correctly annotated in all three cases tested. Finally, the DI-HRMS method reported by Haijes et al. [11] could make a precise diagnosis in 19 of the 21 IEM tested in plasma but did not identify methylenetetrahydrofolate reductase deficiency and carnitine palmitoyltransferase I deficiency. Several causes may explain the lack of abnormal test results and the failure to establish a diagnosis. First, due to the rarity of IEM, some samples used in our method evaluation, as well as in studies by others [8,9,10,11], were taken from patients who had already received specific treatment for their condition, which has resulted in less pronounced or even normalized disease-specific biochemical abnormalities. It is to be expected that undiagnosed and untreated patients will show larger deviations in metabolite patterns, which will improve diagnostic accuracy. Prospective studies are required to demonstrate the full capability of untargeted metabolomics platforms in screening for IEM. Second, technical limitations in the used platforms, such as sample preparation, chromatography, raw data processing and data analysis, may hinder correct test results for certain metabolites [8,9,11]. We expect that developments in data analysis will lead to improvement in the methodology. In raw data processing, for example, better procedures for peak-picking and deconvolution of adducts and isotope signals will improve metabolite identification (our data and [9]). The application of comprehensive databases containing all (possible) biomarkers of IEM, informative metabolite ratios and algorithms assisting in recognition of characteristic metabolite patterns are required to further optimize diagnostic performance. As an example, we show that expanding the number of biomarkers for hyperargininemia facilitates its diagnosis.

It is worth reiterating that comparison of the performance of the different methods reported for IEM screening by untargeted metabolomics platforms is complicated, since many variations exist between the different studies, e.g., different IEM were tested and for each disorder distinct samples with different degrees of metabolite abnormalities were used. Appropriate testing of method performance should be performed by sample exchange programs or External Quality Assurance schemes.

In this study, we describe a novel metabolomics method applying a semi-automatic sample preparation procedure combined with UHPLC-Orbitrap-MS. Our metabolomics platform differentiates signatures of many different IEM from that of controls. The use of metabolomics in the field of IEM diagnostics is still in its childhood and likely has not reached its full potential yet. Nevertheless, our results and those of others [8,9,11] show that the number of IEMs detected by metabolomics increases. Progress in automation, like we applied in our novel method, and data analyses, will make diagnostics of IEM faster and cheaper. We envision that in the near future the current approach to diagnose IEM, with numerous targeted tests, will be replaced by untargeted metabolomics methods. Noticeably, we, as well as others [8,9,11], show the potential of metabolomics in IEM diagnostics, but in none of these reports full analytical and clinical validation has been performed, while this is a requirement before application in the clinic. In addition to the potential of screening for known IEMs, untargeted metabolomics platforms allow the identification of new biomarkers, useful to establish diagnoses or to use as a surrogate marker for disease outcome [9,25,26]. Finally, metabolomics provides a comprehensive biochemical phenotype that facilitates interpretation of possible biochemical consequences of variants of unknown significance identified in whole exome sequencing or whole genome sequencing [9,11,27].

## 4. Materials and Methods

### 4.1. Reagents and Chemicals

Acetonitrile (hypergrade) and formic acid were from Merck (Amsterdam, The Netherlands) and UPLC water and methanol from Biosolve (Valkenswaard, The Netherlands). Internal and external standards (mostly isotope labeled) were selected on the basis of two criteria. First, compounds were selected to represent a number of different compound classes, i.e., amino acids, organic acids, acylcarnitines, purines and pyrimidines and a bile acid. Second, from these different compound classes, compounds were selected to have retention times across the chromatogram. An internal standards mixture was prepared containing 600 µmol/L L-phenylalanine (ring-[D_5_], 98%), 300 µmol/L thymidine (methyl-[^13^C], 98%), 300 µmol/L uracil (1,3-[^15^N], 98%), 500 µmol/L isoleucine ([D_10_], 98%), 225 µmol/L ornithine (3,3,4,4,5,5–[D_6_], 98%), 230 µmol/L tyrosine (ring-[D_4_], 98%) (Cambridge Isotopes Laboratories, Tewksbury, MA, USA), 85 µmol/L 5-bromo-DL-tryptophan, 300 µmol/L 3,3-dimethylglutaric acid, 44 µmol/L glycochenodeoxycholic-2,2,4,4-[D_4_] acid (Sigma Aldrich, Zwijndrecht, The Netherlands), and 285 µmol/L [D_3_]-carnitine (H. ten Brink, Amsterdam UMC, Amsterdam, The Netherlands). An external standards mixture consisted of 100 µmol/L methylmalonic acid (methyl-[D_3_], 98%), 207 µmol/L uridine (ribose-5,5-[D_2_], 98%), 670 µmol/L L-valine ([D_8_], 98%) (Cambridge Isotopes Laboratories, Tewksbury, MA, USA), 45 µmol/L [D_2_]-acetylcarnitine, 21 µmol/L [D_3_]-hexanoylcarnitine, 19 µmol/L [D_3_]-tetradecanoylcarnitine, 6 µmol/L [D_3_]-hexadecanoylcarnitine (H. ten Brink, Amsterdam UMC, Amsterdam, The Netherlands).

Standard mixtures used for external calibration of the Q Exactive Plus were Calmix positive and Calmix negative, for the positive and negative ion mode, respectively (Thermo Fisher Scientific, Breda, The Netherlands). Additionally, an in-house ‘Metabolic Laboratory’ negative ion mode calibration mix was made to ensure mass accuracy for ions with a *m*/*z* < 262, containing 500 µmol/L L-phenylalanine, 500 µmol/L methylmalonic acid, 150 µmol/L taurocholic acid and 330 µmol/L uridine (Sigma Aldrich, Zwijndrecht, The Netherlands). The lock mass solution consisted of 400 mg/L caffeine and 400 mg/L 5-bromo-uracil (Sigma Aldrich, Zwijndrecht, The Netherlands). Custom calibration mix negative ion mode was made by combining 30 µL lock mass solution, 30 µL Metabolic Laboratory negative ion mode calibration mix and 300 µL Calmix negative. The ClinCal amino acid calibrator from Recipe (Munich, Germany) was used as a QC sample.

### 4.2. Sample Selection

Our metabolomics workflow was tested on a range of 33 distinct IEM. In total, 53 plasma samples from 33 known IEM patients (1–4 samples per IEM) were analyzed. IEM diagnoses were previously confirmed by enzyme and/or molecular testing when appropriate. Control samples were obtained from remaining material of patients, which screened negative for all known IEM. Heparin blood samples of both groups were drawn for routine metabolic screening or therapy monitoring without applying a specific protocol on collection of material (e.g., time, fasting/dietary status, treatment). In agreement with national legislation and institutional guidelines, all patients or their guardians approved the possible anonymous use of the remainder of their samples for method validation purposes. The study was conducted in accordance with the Declaration of Helsinki. Samples were stored in a digital-alarm-controlled freezer at −20 °C before analysis for a period ranging from 2 weeks to 14 years. Samples were analyzed in eight experimental runs (batches), including 2–14 IEM samples and 25–38 controls (random age and gender) per batch.

### 4.3. Semi-Automated Sample Preparation Procedure

Semi-automated sample preparation was performed on a Hamilton Robotics ML-STAR eight channel pipetting robot equipped with a camera, iSwap robotic hand, an orbital shaker, an orbital heater/shaker and a vacuum station (Bonaduz, Switzerland) according to the following procedure. All samples were thawed at room temperature for 20–30 min and mixed by vortexing. For each sample a 200 µL aliquot was pipetted into a 1.5 mL polypropylene tube with a unique 2D-barcode containing the sample identifier. Subsequently, all samples were centrifuged at 13,000 rpm for 5 min at 4 °C.

An input file for the pipetting robot was created, containing the name of each sample, the corresponding 2D barcode and whether the sample should be treated as a patient or non-patient sample (e.g., control, blank). Thereafter, the 2D barcodes of the samples in the sample trays were scanned. If the order in the sample trays matched the order on the worklist, sample preparation started. First the vacuum station was assembled by iSwap. Then, 450 µL acetonitrile containing 1% formic acid was added to each well of a Phree 96 well plate (Phenomenex, Maarsen, The Netherlands), which was placed on the shaker. Subsequently 20 µL of internal standards mix was added, followed by 50 µL of sample. For each sample marked as a patient, this was done in triplicate (three separate wells). The 96 well plate was shaken during 2 min at 1000 rpm. After shaking, the iSwap moved the Phree plate to the vacuum station for filtration. A delta pressure of 600 mbar was applied for 5 min to separate the metabolite extract from the protein precipitate. After filtration, the iSwap moved the Phree plate back to the shaker where 500 µL methanol + 1% formic acid was added to each well. The plate was shaken for 2 min at 800 rpm and moved back to the vacuum station for filtration (delta pressure 600 mbar for 5 min) to facilitate extraction of remaining metabolites. The collected filtrate was evaporated to dryness at 60 °C on a Porvair Ultravap (Porvair Sciences Limited, Norfolk, UK). The plate was then placed on the heated shaker position of the pipetting robot and 200 µL water/methanol (95:5) with 0.5% formic acid was added to each well, followed by shaking at 800 rpm for 2 min. Subsequently, 20 µL external standards mix was added to each well of a 350 µL microtiter plate and 130 µL of each sample extract was transferred to the microtiter plate. After shaking for 2 min at 200 rpm, the plate was sealed with a pre-slit PTFE cover (Thermo Scientific, Breda, The Netherlands) and placed in the autosampler of a Dionex Ultimate 3000 UHPLC chromatographic system (Thermo Fisher Scientific, Breda, The Netherlands). Sample preparation and the start of the UHPLC-Orbitrap-MS(/MS) analysis always took place on the same day.

### 4.4. UHPLC-Orbitrap-MS(/MS) Analysis

UHPLC-MS(/MS) analysis was performed using a Dionex Ultimate 3000 UHPLC chromatographic system combined with a Q Exactive Plus mass spectrometer fitted with a heated electrospray source operated in the positive or negative ion mode. The software interface was Xcalibur 4.0.27.42, SII 1.3 and MSTune 2.8 SP 1 (Thermo Fisher Scientific, Breda, The Netherlands).

UHPLC separation was performed on a Kinetex F5 2.6 µm 2.1 mm × 150 mm column equipped with an F5 guard column (Phenomenex, Maarsen, The Netherlands). The column was kept at 20 ± 0.1 °C during analysis. Mobile phases were A: 100% water, 0.5% formic acid also containing 40 µg/L caffeine and 40 µg/L 5-bromo-uracil as lock mass compounds, and B: 100% ACN and 1.0% formic acid. The injection volume for all separations was 3 µL. Chromatographic elution was achieved under gradient conditions with a flow rate of 400 µL/min. Elution started with an isocratic step of 1.03 min at 0% B, followed by a linear gradient from 0% to 25% B (1.03–2.60 min), 25% to 35% B (2.60–5.70 min), and 35% to 95% B (5.70–7.78 min). These conditions were maintained for 3.59 min before returning to 0% B in 0.03 min and equilibration at start conditions for 3.66 min. The total runtime was 15 min.

The Q Exactive Plus mass spectrometer was operated with a capillary voltage of -3.50 kV in the negative ionization mode and 3.50 kV in the positive ionization mode. The capillary temperature was set at 380 °C, and auxiliary gas temperature at 300 °C. The sheath gas pressure, auxiliary gas pressure and sweep gas flow rate were set at 60, 20, and three arbitrary units, respectively, with nitrogen gas. Detection was achieved in both ionization modes from 70 to 1050 *m*/*z*. To set the correct pre-scan frequency, chromatographic peak width (FWHM) was set at 3 s in all modes. In full scan mode, the resolution of the analyzer was set at 140,000 (m/Δm, FWHM @ 200 *m*/*z*). The maximum inject time was set at 100 ms. AGC-target was set to 3E6. In Full scan - ddMS2 mode: The survey scan was obtained with a resolution of 70.000 (m/Δm, FWHM @ 200 *m*/*z*). Maximum inject time was set to 100 ms and AGC-target was set at 3E6. MS/MS scans were acquired with a resolution of 17,500 (m/Δm, FWHM @ 200 *m*/*z*) with a maximum inject time of 35 ms. ddMS settings were as follows: minimum AGC Target: 5.00e3, intensity threshold: 1.4E5, apex trigger: 2 to 4 s and dynamic exclusion: 2.5 s. The AGC-target was set at 2E4. The isolation window was 1.5 *m*/*z*.

Three types of ddMS/MS experiments were performed on a pooled sample containing an aliquot of all patient samples present in the analytical run:

1. Inclusion list set at on (do not pick others)
2. Inclusion list set at on (do pick others), exclusion list set at on
3. Inclusion list set at off, exclusion list set at on

In these experiments, the 12 most abundant ions were fragmented (TopN 12) with an NCE value of 20%, 35% and 50%, unless a specific known optimal NCE value was specified in the inclusion list. These optimal NCE values were taken from the mzCloud website [28]. The inclusion list consisted of 743 small metabolites, many of which were known markers of IEM. The positive and negative ion mode had separate inclusion lists. The ESI(+) inclusion list contained *m*/*z*-values of the [M+H] adducts of all biomarkers. The ESI(−) inclusion list contained m/z-values of the [M−H] adducts.

Prior to each analytical run, the mass spectrometer system performance was evaluated and calibrated using Calmix positive for the positive ion mode using the predefined evaluation and calibration of the manufacturer, including mass-calibration on *N*-tert-butylamine, caffeine and MRFA to ensure mass accuracy for ions <200 *m*/*z*. For the negative ion mode Calmix negative was used. System evalution and calibration were performed using the predefined evaluation and calibration options of the manufacturer. Subsequently, a custom mass-calibration was performed with our in-house ‘Metabolic Laboratory’ negative ion mode calibration mix to ensure mass accuracy for ions <262 *m*/*z*.

During analysis lock-mass calibration was used to maximize mass accuracy. Caffeine and 5-bromo-uracil were used as lock masses, for the positive and negative ion mode, respectively.

Generation of the LC-MS sequence list was integrated in the semi-automatic sample preparation. This enabled random injection order of the patient and control samples. Each sequence of UHPLC-Orbitrap-MS analyses was started with the positive ion mode. The UHPLC start-up method was followed by four injections of the blank sample (water) to stabilize the chromatographic column. Subsequently, the QC sample was measured. Thereafter, the patient and control samples were analyzed in a random order, with an analysis of the QC sample after every 10th run. When all patient and control samples were measured, DDA-MS/MS analyses were performed. Then, all samples were measured in the negative ion mode. The negative ion mode started with four injections of the blank. The same procedure was followed, as described for the positive ion mode. The DDA-MS/MS analyses in the negative ion mode were followed by a shutdown program.

### 4.5. Quality Control

#### 4.5.1. Retention Time Stability

Retention time of all standards was monitored in all plasma-based samples. The following tolerances were applied for all standards. The maximum allowed within-batch RT variation was set at <1.5% and the maximum allowed between batch RT variation was set at <2%.

#### 4.5.2. Within Batch Peak Area Variation

Peak areas of the internal and external standards in all plasma-based samples were integrated using Xcalibur 4.0 and manually reviewed in the Quan-browser module of Xcalibur. [D_5_]-phenylalanine, 1,3-[^15^N]-uracil, [D_10_]-isoleucine, [D_6_]-ornithine, [D_4_]-tyrosine, 5-bromo-DL-tryptophan, 3,3-dimethylglutaric acid, [D_3_]-carnitine, [D_2_]-uridine, [D_8_]-valine, [D_2_]-acetylcarnitine, [D_3_]-hexanoylcarnitine, [D_3_]-tetradecanoylcarnitine, [D_3_]-hexadecanoylcarnitine were monitored in positive ion mode and [D_5_]-phenylalanine, [^13^C]-thymidine, 1,3-[^15^N]-uracil, [D_4_]-tyrosine, 5-bromo-DL-tryptophan, 3,3-dimethylglutaric acid, [D_4_]-glycochenodeoxycholic acid, [D_3_]-methylmalonic acid, [D_2_]-uridine, and [D_8_]-valine were monitored in the negative ion mode. The within-run CV of the peak area of all standards was not allowed to exceed 30%.

Additionally, in the QC sample, the CVs in the peak area of all annotated endogenous metabolites were calculated. Then the calculated CVs were binned on retention time (increments of 1.0 min) and subsequently the median of each bin was taken.

#### 4.5.3. Between-Batch Peak Area Variation

The median peak area of all internal and external standards within the measured QC samples was monitored. The between-batch variation was not allowed to exceed 30%.

#### 4.5.4. Data Processing

The raw data were imported into Progenesis QI v2.4 (Newcastle-upon-Tyne, UK). Progenesis QI deals with alignment of the chromatograms, normalization, deisotoping, adduct deconvolution, peak picking and peak annotation. All settings used within Progenesis QI can be found in the supplementary materials (S7). Progenesis QI, builds an aggregate of the features detected in all samples included in a batch. Any feature detected in one sample in a batch will be detected in all samples in that batch. Each batch contained different samples of patients with varying IEM. Therefore, the number of annotated metabolites varied per batch due to the presence of abnormal metabolites only detected in patient samples. Initially, HMDB 4.0 was used for compound annotation [29,30,31,32]. Compound ions were annotated by matching the retention time (max. Δ RT: 0.15 min), isotope pattern similarity (>85%) and m/z-value of a feature (max. Δ ppm: 3) with an in-house database containing metabolites which are known biomarkers for IEM. At the time of writing, this database contains 757 entries of endogenous metabolites, of which 408 have a retention time validated by standards or plasma samples of IEM patients with established metabolite abnormalities. Known co-eluting isomeric compounds share one entry in the database. A selection of 339 metabolites relevant to IEM screening was used to annotate compound ions for routine diagnostic purposes (list available on request). MS/MS spectra were included when available to increase the confidence of the annotation. Here, the dot product score must be larger than 0.60. The confidence level of metabolite annotation was established according to the MSI initiative reporting standard [17].

After these processing steps, we obtained for every batch a matrix containing abundancies where every element corresponded to a sample and feature. Note that some abundancies are the summation of the detected adducts and their isotopes. This matrix, together with qualitative data (e.g., ppm error, isotopic pattern and annotation), was exported as a csv-file and processed by our data pipeline. Within the pipeline, z-scores were calculated for each metabolite by using 15 control samples originating from the same batch as the patient. The z-score was defined as the number of standard deviations a measured value was above or below the mean of the control group. Matching of the controls with the patient was performed on age and sex. First, the controls with the same sex were selected, then we determined the most age-related controls and defined an age cut-off based on the following equation, where age is in years:

$${\mathrm{age}}_{\mathrm{patient}}{}^{0.95}-0.5\leq {\mathrm{age}}_{\mathrm{control}}\leq {\mathrm{age}}_{\mathrm{patient}}{}^{1.05}+0.5$$

These cut-offs have the tendency to be less strict for increasing age and have a small bias towards older reference samples. When there were less than 15 controls fulfilling these conditions, additional controls were chosen by their similarity in age (which might include the opposite sex). Note that, because of the limited number of controls in a batch, this matching was also limited.

Technical triplicates of patient samples allowed us to gain insight into the technical variability of every metabolite. Excessive technical variability was detected by using the Welch’s *t*-test, which was considered appropriate since the variance of the triplicate differed from the variance of the reference population (the 15 controls). We expected triplicates with a relatively large variance but distant average from the reference average to have low p-values (acceptable measurement). Increasing the variance of the triplicate while fixing the distance to the reference average should lead to increasing *p*-values (unacceptable measurement). For *p*-value >0.05, we considered that technical variability of the triplicate was too large to rely on the inferred z-score (average of the triplicates).

## 5. Conclusions

In this paper we describe a novel untargeted metabolomics platform for screening IEM combining semi-automatic sample preparation with pentafluorophenylpropyl phase (PFPP)-based UHPLC-Orbitrap-MS. We demonstrate robust performance and show that our method differentiates metabolite patterns of many different IEM from those of controls.

## Figures and Tables

**Table 1:** Method evaluation for 33 distinct inborn errors of metabolism (IEM).

Inborn Error of Metabolism ^a^	Z-Score ^b^	*p*-Value ^c^	ID ^d^
**2-Methyl-3-hydroxybutyryl-CoA dehydrogenase deficiency (N = 1)**			
2-Methyl-3-hydroxybutyric acid [↑] (+/−)	NF	NF	-
Tiglylglycine [↑] (−)	7.0 *	2.99 × 10^−3^	2b
**3-Methylcrotonyl-CoA-carboxylase deficiency (N = 1)**			
3-Hydroxyisovaleric acid [↑] (+/−)	NF	NF	-
3-Methylcrotonylglycine [↑] (−)	33.7 *	1.50 × 10^−2^	2b
3-Hydroxyisovalerylcarnitine [↑] (+)	1811.7 *	4.35 × 10^−3^	2b
**Adenylosuccinate lyase deficiency (N = 1)**			
SAICAR [↑] (+/−)	NF	NF	-
Succinyladenosine [↑] (−)	57.2 *	2.80 × 10^−3^	2b
**Alkaptonuria (N = 1)**			
Homogentisic acid [↑] (+)	0.6	3.78 × 10^−1^	2b
**Alpha-Methylacyl-CoA racemase deficiency (N = 2)**			
Pristanoyl-carnitine [↑] (+/−)	NF	NF	-
Phytanoyl-carnitine [↑] (+/−)	NF	NF	-
Dihydroxycholestanoic acid + Gly [↑] (−)	INF/INF	<1.1 × 10^−16^/<1.1 × 10^−16^	2b
Dihydroxycholestanoic acid + Tau [↑] (−)	121.5 */34.1 *	1.95 × 10^−3^/1.51 × 10^−2^	2b
Trihydroxycholestanoic acid + Gly [↑] (−)	462.6 */386.6 *	1.70 × 10^−3^/1.45 × 10^−2^	2b
Trihydroxycholestanoic acid + Tau [↑] (−)	500.4 */326.5 *	9.07 × 10^−4^/1.70 × 10^−2^	2b
**Aminoacylase I deficiency (N = 1)**			
*N*-Acetylalanine [↑] (−)	8.7 *	1.74 × 10^−2^	2b
*N*-Acetylarginine [↑] (−)	0.4	1.69 × 10^−1^	2b
*N*-Acetylglutamic acid [↑] (−)	362.9 *	1.19 × 10^−2^	2b
*N*-Acetylglycine [↑] (−)	8.9 *	1.06 × 10^−2^	2b
*N*-Acetylleucine [↑] (−)	23.0 *	1.31 × 10^−2^	2b
*N*-Acetylmethionine [↑] (−)	644.7 *	8.44 × 10^−3^	2b
*N*-Acetylserine [↑] (−)	NF	NF	-
*N*-Acetylthreonine [↑] (+)	1259.4 *	1.31 × 10^−2^	2b
**Arginase deficiency (N = 1)**			
2-Oxoarginine [↑] (−)	NF ^e^	NF	-
4-Guanidinobutyric acid [↑] (−)	25.7 *	1.18 × 10^−2^	2b
Arginine [↑] (+)	2.4	5.14 × 10^−2^	1
Glutamine + Glutamic acid [↑] (−)	−0.1	7.51 × 10^−1^	1
Guanidinoacetic acid [↑] (−)	2.7 *	6.08 × 10^−6^	1
Homoarginine [↑] (+)	9.7 *	3.45 × 10^−2^	2b
*N*-Acetylarginine [↑] (−)	137.2 *	3.20 × 10^−4^	2b
Orotic acid [↑] (−)	32.4 *	5.85 × 10^−4^	2b
Uracil [↑] (−)	5.7 *	4.02 × 10^−3^	1
Uridine [↑] (−)	3.1 *	1.60 × 10^−6^	1
Argininic acid [↑] (+/−)	NF ^e^	NF	-
**Argininosuccinic aciduria (N = 3)**			
Arginine [↓] (+)	−0.9 *–−0.3	2.01 × 10^−1^–2.98 × 10^−2^	1
Argininosuccinic acid [↑] (−)	4797 *–13,725 *	2.96 × 10^−2^–5.40 × 10^−3^	2b
Citrulline [↑] (−)	10 *–32 *	4.87 × 10^−3^–2.83 × 10^−4^	1
Cytidine [↑] (−)	3580 */2.1 */NF	9.78 × 10^−3^/7.33 × 10^−6^/NF	1
Glutamine + Glutamic acid [↑] (+)	2.2 */−1.8 */NF	6.99 × 10^−3^/7.51 × 10^−6^/NF	1
Homocitrulline [↑] (+)	1.7 *–4.5	6.69 × 10^−4^–2.81 × 10^−5^	2b
*N*-Acetylcitrulline [↑] (−)	6.2 *–42.7 *	8.23 × 10^−3^–9.02 × 10^−4^	2b
Orotic acid [↑] (−)	0.1/−0.5/NF	6.61 × 10^−1^/1.08 × 10^−1^/NF	2b
Uracil [↑] (−)	−0.7 */−1.9 * /NF	1.89 × 10^−2^/5.20 × 10^−6^/NF	1
Uridine [↑] (−)	−0.4–3.4 *	1.70 × 10^−1^–1.06 × 10^−3^	1
**Beta-ketothiolase deficiency (N = 2)**			
2-Methyl-3-hydroxybutyric acid [↑] (−)	5.8 */15.7 *	2.66 × 10^−2^/1.03 × 10^−3^	4
2-Methylacetoacetic acid [↑] (−)	0.5/NF	5.84 × 10^−1^/NF	4
Tiglylcarnitine [↑] (+)	0.3/NF	0.726/NF	2b
Tiglylglycine [↑] (−)	190 */217 *	1.23 × 10^−2^/7.96 × 10^−5^	2b
**Beta-mannosidosis (N = 1)**			
GlcNAc-Man [↑] (−)	383.9 *	5,73 × 10^−3^	2b
**Carbamoyl phosphate synthetase I deficiency (N = 2)**			
Glutamine + Glutamic acid [↑] (+)	4.7 */0.59	6.43 × 10^−5^/1.4 × 10^−1^	1
**Citrullinemia type I (N = 1)**			
Arginine [↓] (+)	0.5	4.06 × 10^−1^	1
Citrulline [↑] (+)	128.7 *	3.64 × 10^−3^	1
Citrulline lactam [↑] (+)	202,9	9.13 × 10^−2^	4
Glutamine + Glutamic acid [↑] (+)	−2.4 *	3.28 × 10^−2^	1
*N*-Acetylcitrulline [↑] (−)	335.2 *	3.77 × 10^−3^	2b
Orotic acid [↑] (−)	0.3	3.64 × 10^−1^	2b
Uracil [↑] (−)	0.1	8.50 × 10^−1^	1
Uridine [↑] (−)	1.4 *	6.41 × 10^−3^	1
**Glutamate formiminotransferase deficiency (N = 1)**			
Formiminoglutamic acid [↑] (+)	2031.0 *	7.13 × 10^−3^	2b
Hydantion-5-propionic acid [↑] (−)	12.0 *	2.24 × 10^−5^	2b
**Glutaric aciduria I (N = 2)**			
3-Hydroxyglutaric acid [↑] (−)	2.1 */NF	5.49 × 10^−5^/NF	4
Glutarylcarnitine [↑] (+)	317.4 */338.6 *	5.57 × 10^−3^/4.72 × 10^−3^	1
Glutaric acid [↑] (−)	0.7/2.5 *	3.05 × 10^−5^/2.16 × 10^−7^	1
Glutarylglycine [↑] (−)	228.8 */513.4 *	2.90 × 10^−3^/9.19 × 10^−4^	4
**Glutaric aciduria II (N = 2)**			
2-Hydroxyglutaric acid [↑] (−)	39.1 */73.7 *	6.27 × 10^−3^/2.23 × 10^−3^	4
3-Hydroxyglutaric acid [↑] (−)	2.9 */NF	2,15 × 10^−5^/NF	4
Ethylmalonic acid [↑] (−)	1.2/53.6 *	1.61 × 10^−1^/5.02 × 10^−3^	1
Glutaric acid [↑] (−)	1.8 */2.1 *	9.31 × 10^−6^/3.03 × 10^−5^	1
Hexanoylglycine [↑] (−)	391.2 */1797.4 *	6.37 × 10^−3^/1.05 × 10^−3^	2b
Isobutyrylglycine [↑] (−)	16.4 */NF	4,56 × 10^−3^/NF	2b
Isovaleryglycine [↑] (−)	51.5 */1.1 *	7.78 × 10^−3^/3.37 × 10^−2^	2b
(Iso)butyrylcarnitine [↑] (+)	30.2 */110.2 *	5.65 × 10^−4^/8.95 × 10^−4^	1
Isovalerylcarnitine [↑] (+)	189.6 */34.1 *	9.08 × 10^−4^/3.06 × 10^−3^	1
Glutarylcarnitine [↑] (+)	28.7 */85.9 *	2.37 × 10^−3^/9.32 × 10^−4^	2b
Hexanoylcarnitine [↑] (+)	124.5 */184.6 *	3.24 × 10^−3^/9.02 × 10^−3^	1
Octanoylcarnitine [↑] (+)	101.8 */123.1 *	2.71 × 10^−3^/6.21 × 10^−4^	1
Decanoylcarnitine [↑] (+)	72.0 */70.4 *	1.73 × 10^−3^/1.47 × 10^−3^	1
Dodecanoylcarnitine [↑] (+)	0.9/80.0 *	7.21 × 10^−2^/1.01 × 10^−2^	1
Tetradecanoylcarnitine [↑] (+)	125.7/101.7 *	1.51 × 10^−1^/5.78 × 10^−3^	1
Tetradecenoylcarnitine [↑] (+)	100.8 */61.9 *	4.70 × 10^−2^/2.82 × 10^−4^	1
Hexadecanoylcarnitine [↑] (+)	6.1/9.9 *	1.99 × 10^−1^/1.97 × 10^−2^	1
Hexadecenoylcarnitine [↑] (+)	189.3/130.2 *	1.16 × 10^−1^/5.25 × 10^−3^	1
Octadecanoylcarnitine [↑] (+)	4.2/4.5	1.80 × 10^−1^/1.43 × 10^−1^	1
Oleoylcarnitine [↑] (+)	7.5/11.8 *	1.87 × 10^−1^/4.68 × 10^−2^	1
Linoleoylcarnitine [↑] (+)	13.9/21.5 *	1.54 × 10^−1^/1.09 × 10^−3^	1
**Homocystinuria (CBS deficiency) (N = 3)**			
Cysteinyl-homocysteine [↑] (+/−)	NF	NF	-
Homocysteine [↑] (+)	0.5–1.2 *	9.93 × 10^−2^–4.93 × 10^−3^	1
Homocystine [↑] (+/−)	NF	NF	-
Methionine + Methionine sulfoxide [↑] (+)	6.5 *–55.1 *	5.01 × 10^−3^–2.71 × 10^−4^	1
Homocysteic acid [↑] (+/−)	NF	NF	-
**Isovaleric acidemia (N = 1)**			
3-Hydroxyisovaleric acid [↑] (−)	−1.8 *	8.52 × 10^−5^	1
4-Hydroxyisovaleric acid [↑] (−)	NF	NF	-
Isovalerylcarnitine [↑] (+)	69.2 *	1.40 × 10^−4^	1
Isovaleryglycine [↑] (−)	159.9 *	8.82 × 10^−4^	1
**Long-chain-3-hydroxyacyl CoA dehydrogenase deficiency (N = 2)**			
3-Hydroxyadipic acid [↑] (−)	NF	NF	-
3-Hydroxydecanedioic acid [↑] (−)	NF	NF	-
3-Hydroxytetradecenoylcarnitine [↑] (+)	11.7 */19.6 *	8.84 × 10^−3^/2.56 × 10^−3^	2b
3-Hydroxytetradecanoylcarnitine [↑] (+)	37.9 */16.9 *	1.90 × 10^−2^/3.54 × 10^−2^	2b
3-Hydroxyhexadecenoylcarnitine [↑] (+)	142.4 */61.6 *	2.39 × 10^−2^/2.94 × 10^−2^	2b
3-Hydroxyhexadecanoylcarnitine [↑] (+)	402.1/67.3	8.01 × 10^−2^/1.62 × 10^−1^	2b
3-Hydroxyoleoylcarnitine [↑] (+)	1141.8/149.3	7.30 × 10^−2^/1.56 × 10^−1^	2b
3-Hydroxyoctadecanoylcarnitine [↑] (+)	754.6/35.2	9.32 × 10^−2^/1.93 × 10^−1^	2b
Sebacic acid [↑] (−)	6.1 */2.3 *	1.92 × 10^−2^/5.01 × 10^−3^	4
Suberic acid [↑] (−)	2.2/1.6 *	2.06 × 10^−1^/3.44 × 10^−2^	4
**Lysinuric protein intolerance (N = 2)**			
Arginine [↓] (+)	−1.5 */2.4 *	6.40 × 10^−3^/3.04 × 10^−6^	1
Glutamine + Glutamic acid [↑] (+)	3.2 */6.4 *	3.46 × 10^−4^/1.83 × 10^−13^	1
Lysine [↓] (+)	−1.6 */−1.9 *	2.50 × 10^−5^/1.38 × 10^−5^	1
Ornithine [↓] (+)	−2.1 */1.1	5.87 × 10^−1^/3.41 × 10^−6^	1
Orotic acid [↑] (−)	4.0 */NF	3.27 × 10^−2^/NF	4
**Malonyl-CoA decarboxylase deficiency (N = 1)**			
Malonylcarnitine [↑] (+)	132.7 *	9.84 × 10^−3^	2b
Malonic acid [↑] (−)	0.1	8.39 × 10^−1^	1
**Maple syrup urine disease (N = 2)**			
(allo)Isoleucine [↑] (+)	3.1 */17.7 *	3.91 × 10^−3^/4.95 × 10^−3^	1
2-Hydroxy-3-methylbutyric acid [↑] (−)	NF	NF	-
2-Hydroxy-3-methylvaleric acid [↑] (−)	NF	NF	-
2-Hydroxy-4-methylvaleric acid [↑] (−)	NF	NF	-
2-Keto-3-methylbutyric acid [↑] (−)	NF	NF	-
2-Keto-3-methylvaleric acid [↑] (−)	−1.9 */25.5 *	2.95 × 10^−6^/4.52 × 10^−2^	4
2-Keto-4-methylvaleric acid [↑] (−)	−2.0 */1.4	1.45 × 10^−6^/1.48 × 10^−1^	4
Leucine [↑] (+)	3.1 */22.1 *	6.85 × 10^−3^/4.25 × 10^−3^	1
Valine [↑] (+)	1.2/5.2 *	5.61 × 10^−2^/4.63 × 10^−3^	1
**Medium chain acyl-CoA dehydrogenase deficiency (N = 4)**			
5-Hydroxyhexanoic acid [↑] (−)	NF	NF	-
7-Hydroxyoctanoic acid [↑] (−)	NF	NF	-
Decenoylcarnitine [↑] (+)	8.8 *–45.1 *	4.24 × 10^−3^–6.42 × 10^−5^	1
Hexanoylcarnitine [↑] (+)	11.2 *–54.3 *	2.12 × 10^−3^–1.11 × 10^−5^	1
Octanoylcarnitine [↑] (+)	25.5 *–60.7 *	4.63 × 10^−3^–1.87 × 10^−3^	1
Decenedioic acid [↑] (−)	NF	NF	-
Hexanoic acid/*Trans*-cyclohexan × 10-1,2-diol [↑] (−)	−0.4–1.9	6.69 × 10^−1^–2.51 × 10^−1^	1
Hexanoylglycine [↑] (−)	29.3 *–125.4 *	1.23 × 10^−2^–6.83 × 10^−3^	2b
Octanoic acid [↑] (−)	26.5 *–61.5 *	9.37 × 10^−3^–2.57 × 10^−3^	4
Octanoylglycine [↑] (−)	169.5 *–1281.0	9.76 × 10^−3^–2.02 × 10^−3^	2b
Phenylpropionylglycine [↑] (−)	220.0 *–1629.8 *	9.89 × 10^−3^–2.44 × 10^−3^	2b
Sebacic acid [↑] (−)	0.0–−0.3	9.94 × 10^−1^–3.90 × 10^−1^	4
Suberic acid [↑] (−)	−0.1–0.6 *	8.34 × 10^−1^–3.44 × 10^−2^	4
Suberylglycine [↑] (−)	61.3 *–350.5 *	1.90 × 10^−2^–3.83 × 10^−3^	2b
Undecanoylcarnitine [↑] (+)	−0.6–0.1	7.88 × 10^−1^–9.32 × 10^−2^	4
Heptanoylcarnitine [↑] (+)	3.1 *–10.7 *	2.13 × 10^−2^–6.56 × 10^−4^	4
Nonanoylcarnitine [↑] (+)	53.7 *–110.5 *	4.37 × 10^−3^–8.91 × 10^−4^	4
**Methylmalonyl-CoA mutase deficiency (N = 1)**			
Propionylcarnitine [↑] (+)	102.2 *	1.91 × 10^−3^	1
Methylmalonylcarnitine [↑] (+)	2201.5 *	1.51 × 10^−2^	1
Methylcitric acid (1) [↑] (−)	1204.5 *	1.12 × 10^−3^	1
Methylcitric acid (2) [↑] (−)	4.5 *	3.41 × 10^−5^	1
Methylmalonic acid [↑] (−)	221.5 *	2.81 × 10^−3^	1
**Mevalonic aciduria (N = 1)**			
Mevalonic acid [↑] (−)	0.9	9.00 × 10^−2^	4
Mevalonolactone [↑] (−)	NF	NF	-
**Organic cation transporter 2 deficiency (N = 1)**			
Carnitine [↓] (+)	−2.2 *	2.39 × 10^−3^	1
**Ornithine aminotransferase (N = 1)**			
3-Amino-2-piperidone [↑] (+)	29.5 *	7.96 × 10^−4^	4
Guanidinoacetic acid [↓] (+)	−1.2 *	2.60 × 10^−4^	1
Ornithine [↑] (+)	31.7 *	2.51 × 10^−3^	1
**Ornithine transcarbamylase deficiency (N = 2)**			
Citrulline [↓] (+)	1.8 */1.8 *	2.09 × 10^−3^/5.18 × 10^−6^	1
Glutamine + Glutamic acid [↑] (+)	1.5 *	7.07 × 10^−5^	1
Orotic acid [↑] (−)	0.2/NF	0.394/NF	4
Uridine [↑] (−)	7.3 */4.8 *	2.36 × 10^−6^/7.07 × 10^−3^	1
**Phenylketonuria (N = 3)**			
2-Hydroxyphenylacetic acid [↑] (−)	NF	NF	1
Glutamylphenylalanine [↑] (+)	64.3 */31.5 */NF	1.30 × 10^−3^/1.44 × 10^−2^/NF	1
*N*-Acetylphenylalanine [↑] (−)	42.7 *–136.2 *	3.33 × 10^−2^–1.35 × 10^−3^	1
*N*-Lactoyl-phenylalanine [↑] (−)	39.9 */41.6 *	2.77 × 10^−2^–9.96 × 10^−3^	1
Phenylacetic acid [↑] (−)	1.7 *–6.0 *	1.01 × 10^−2^–3.03 × 10^−5^	1
Phenylalanine [↑] (+)	29.0 *–88.1 *	9.42 × 10^−3^–<1.1 × 10^−16^	1
Phenylalanylphenylalanine [↑] (+)	10.3 */33.3 */NF	1.10 × 10^−2^/1.21 × 10^−2^ /NF	1
Phenyllactic acid [↑] (−)	71.8 */125.4 */NF	3.33 × 10^−3^/1.04 × 10^−2^/NF	1
Phenylpyruvic acid [↑] (−)	−0.8 */−0.1/NF	8.01 × 10^−3^/7.57 × 10^−1^/NF	1
alpha-*N*-Phenylacetylglutamine [↑] (−)	1.4 *–4.2 *	3.64 × 10^−4^–3.20 × 10^−6^	1
**Propionic acidemia (N = 1)**			
2-Methyl-3-hydroxybutyric acid [↑] (−)	NF	NF	-
3-Hydroxypropionic acid [↑] (−)	NF	NF	-
3-Hydroxyvaleric acid [↑] (−)	NF	NF	-
3-Ketovaleric acid [↑] (−)	NF	NF	-
Propionylcarnitine [↑] (+)	137.4 *	1.13 × 10^−4^	1
Glycine [↑] (+)	14.5 *	5.46 × 10^−3^	1
Methylcitric acid (1) [↑] (−)	1001.9 *	6.26 × 10^−3^	1
Methylcitric acid (2) [↑] (−)	576.1 *	2.84 × 10^−3^	1
Propionylglycine [↑] (−)	950.7 *	1.27 × 10^−3^	2b
Tiglylglycine [↑] (−)	NF ^e^	NF	-
**Thymidine phosphorylase deficiency (N = 1)**			
Deoxyuridine [↑] (−)	370.9 *	6.97 × 10^−4^	1
Thymidine [↑] (−)	64.6 *	2.20 × 10^−3^	1
Thymine [↑] (−)	NF	NF	-
Uracil [↑] (−)	−0.9	1.12 × 10^−1^	1
**Tyrosinemia I (N = 2)**			
4-Hydroxyphenylacetic acid [↑] (−)	140.8 */NF	6.53 × 10^−3^/NF	1
4-Hydroxyphenyllactic acid [↑] (−)	389.5 */1013.8 *	6.02 × 10^−3^/3.39 × 10^−3^	1
4-Hydroxyphenylpyruvic acid [↑] (−)	4.2 */0.6	7.29 × 10^−7^/5.69 × 10^−1^	1
*N*-Acetyltyrosine [↑] (−)	32.4 */96.0 *	9.55 × 10^−3^/5.73 × 10^−3^	1
Phenylpyruvic acid [↑] (+)	113.8 */NF	4.09 × 10^−3^/NF	1
Succinylacetone [↑] (−)	NF	NF	-
Tyrosine [↑] (+)	18.8 */36.9 *	4.82 × 10^−3^/1.88 × 10^−4^	1
**Very long chain acyl-CoA dehydrogenase deficiency (N = 1)**			
Tetradecanoylcarnitine [↑] (+)	36.6 *	6.31 × 10^−3^	2b
Tetradecenoylcarnitine [↑] (+)	85.5 *	1.97 × 10^−2^	2b
Tetradecadienoylcartinine [↑] (+)	42.9 *	1.38 × 10^−2^	2b
Hexadecanoylcarnitine [↑] (+)	6.8 *	9.69 × 10^−3^	1
Octadecanoylcarnitine [↑] (+)	2.7 *	1.06 × 10^−2^	1
Oleoylcarnitine [↑] (+)	5.9 *	4.26 × 10^−5^	2b
**Carnitine palmitoyltransferase II (N = 2)**			
Hexadecanoylcarnitine [↑] (+)	9.6 */21.6 *	9.01 × 10^−3^/2.28 × 10^−2^	1
Octadecanoylcarnitine [↑] (+)	12.6 */33.4 *	1.22 × 10^−2^/2.70 × 10^−2^	1
Oleoylcarnitine [↑] (+)	7.2 */21.4 *	4.10 × 10^−3^/4.06 × 10^−2^	2b
Sebacic acid [↑] (−)	0.2/−0.3	7.59 × 10^−1^/4.69 × 10^−1^	4
Suberic acid [↑] (−)	0.7/−0.1	3.78 × 10^−1^/7.82 × 10^−1^	4

a: For each IEM, the number of patients analyzed is indicated between brackets. For each biomarker the expected change (↑ = elevated, ↓ = decreased) is indicated between square brackets and the ion mode (+ = ESI-(+), − = ESI-(−), +/− = ESI-(+) and ESI-(−)) between brackets for each z-score reported; b: Average z-score of a metabolite in patients, * indicates Welch’s *t*-test on the triplicates yields a *p*-value < 0.05, slash indicates separate values of two patients. Two dashes indicate the range of z-scores when the number of patients ≥ 3, NF = marker not detected or annotated; c: Slash indicates separate *p*-values of two patients. Two dashes indicate the range of the p-values when the number of patients ≥ 3, NF = marker not detected/annotated; d: Confidence level of metabolite annotation according to MSI initiative reporting standard [17]; e: Marker detected in LC-MS analysis, not peak-picked by Progenesis QI.

## Supplementary Materials

The following are available online at https://www.mdpi.com/2218-1989/9/12/289/s1, Table S1: Within-batch variation in retention time, Table S2: Between-batch variation in retention time, Table S3: Within-batch variation in peak area, Table S4: Between-batch variation in peak area, Figure S5: Peak area variation of all metabolites annotated in the QC sample, Figure S6: Separation of the isomeric pairs isoleucine/leucine and betaine/valine, Table S7: Progenesis QI settings, MS Excel file S8: Information on annotated features used for determination of the variation in peak area.
