# Supplementary material for: Untargeted Metabolomics-Based Screening Method for Inborn Errors of Metabolism using Semi-Automatic Sample Preparation with an UHPLC- Orbitrap-MS Platform
Source: Metabolites. 2019 Nov 26;9(12):289. doi: 10.3390/metabo9120289 (PMC6950026; doi:10.3390/metabo9120289)
Supplement: Supplementary file 1 [file metabolites-09-00289-s001.zip › supplementary materials/Supp_paper_metabolites.pdf]

Table S1: Within batch variation in retention time

Monitored compounds ESI (+)	Median CV (%)	Range in CV (%)
3,3 – dimethylglutaric acid	0.08	0.08 - 0.26
5 - bromotryptophan	0.09	0.08 - 0.25
D_2 - acetylcarnitine	0.30	0.22 - 1.12
D_3 - carnitine	0.39	0.21 - 0.79
D_3 - hexadecanoylcarnitine	0.04	0.04 - 0.05
D_3 - hexanoylcarnitine	0.08	0.07 - 0.24
D_{10} - isoleucine	0.24	0.21 - 0.78
D_6 - ornithine	0.47	0.10 - 0.83
D_5 - phenylalanine	0.11	0.08 - 0.37
D_3 - tetradecanoylcarnitine	0.04	0.04 - 0.05
D_4 - tyrosine	0.24	0.16 - 0.74
1,3 - ^{15}N - uracil	0.31	0.29 - 0.78
D_2 - uridine	0.26	0.22 - 0.79
D_8 - valine	0.54	0.52 - 1.12

Monitored compounds ESI (-)	Median CV (%)	Range in CV (%)
3,3 – dimethylglutaric acid	0.08	0.06 - 0.21
5 - bromotryptophan	0.08	0.07 - 0.21
D_4 – glycochenodeoxycholic acid	0.04	0.04 - 0.12
D_{10} - isoleucine	0.25	0.23 - 0.37
D_3 – methylmalonic acid	0.29	0.23 - 0.35
D_5 - phenylalanine	0.11	0.10 - 0.22
^{13}C - thymidine	0.11	0.08 - 0.28
D_4 - tyrosine	0.22	0.14 - 0.28
1,3-^{15}N - uracil	0.33	0.30 - 0.37
D_2 - uridine	0.22	0.20 - 0.32

Table S2: Between batch variation in retention time

Monitored standard	Ion mode	Median RT (min)	CV (%)	CV (%) column 1[a]	CV (%) column 2[b]
D_6 - ornithine	+	0.74	0.55	0.65	0.17
D_3 - carnitine	+	0.91	0.48	0.73	0.08
D_8 - valine	+/-	1.09	1.45	0.74	0.53
1,3-^{15}N - uracil	+/-	1.32	1.55	0.43	0.73
D_{10} - isoleucine	+/-	1.82	3.84	0.74	1.17
D_2 - uridine	+/-	1.96	4.68	0.70	1.70
D_3 – methylmalonic acid	-	2.07	5.32	0.86	2.00
D_2 - acetylcarnitine	+	2.31	1.65	1.36	0.86
D_4 - tyrosine	+/-	2.72	5.35	1.01	1.93
D_5 - phenylalanine	+/-	3.61	1.49	0.29	0.76
^{13}C - thymidine	+/-	3.65	0.97	0.22	0.24
3,3 - dimethylglutaric acid	+/-	4.40	0.80	0.29	0.76
D_3 - hexanoylcarnitine	+	5.04	0.19	0.18	0.22
5 - bromotryptophan	+/-	5.10	0.22	0.22	0.16
D_4 - glycochenodeoxycholic acid	-	8.19	0.14	0.07	0.10
D_3 - tetradecanoylcarnitine	+	8.99	0.34	0.28	0.21
D_3 - hexadecanoylcarnitine	+	9.34	0.46	0.39	0.21

a: CV based on 3 batches, which consist out of 169 injections per ion mode
b: CV based on 5 batches, which consist out of 395 injections per ion mode

Table S3: Within batch variation in peak area

Positive ion mode

ISTD	Median CV (%)	Range CV (%)
D_5 - phenylalanine	13.0	9.8 - 19.1
3,3 – dimethylglutaric acid	12.8	8.8 - 15.8
1,3-^{15}N - uracil	13.8	9.6 - 17.4
D_{10} - isoleucine	13.3	10.2 - 16.7
D_3 - carnitine	13.3	9.1 - 17.4
D_6 - ornithine	16.3	10.7 - 19.3
5 - bromotryptophan	16.3	12.1 - 21.1
D_4 - tyrosine	14.3	10.3 - 17.6

ESTD	Median CV (%)	Range CV (%)
D_8 - valine	7.2	4.3 - 14.9
D_2 - uridine	6.7	5.0 - 11.2
D_2 - acetylcarnitine	8.0	6.4 - 14.5
D_3 - hexanoylcarnitine	6.4	4.5 - 10.1
D_3 - tetradecanoylcarnitine	19.0	13.1 - 30.3
D_3 - hexadecanoylcarnitine	19.4	15.3 - 28

Negative ion mode

ISTD	Median CV (%)	Range CV (%)
D_5 - phenylalanine	14.8	11 - 17.4
^{13}C - thymidine	12.4	9.2 - 16.6
3,3 – dimethylglutaric acid	10.8	8.2 - 15.2
5 - bromotryptophane	14.9	11.6 - 19.7
D_4 - tyrosine_D4	16.2	11.2 - 18.9
D_4 – glycochenodeoxycholic acid	18.4	14.3 - 19.3

ESTD	Median CV (%)	Range CV (%)
D_3 – methylmalonic acid	13.7	6.4 - 18.8
D_2 - uridine	9.3	7.5 - 11.9

Table S4: Between batch variation in peak area

Positive ion mode

ISTD	CV (%)
1,3 - ^{15}N - uracil	19
5 - bromotryptophan	22
D_3 - carnitine	23
D_6 - ornithine	25
D_4 - tyrosine	27
D_{10} - isoleucine	28
D_5 - phenylalanine	29
3.3 - dimethylglutaric acid	68

ESTD	CV (%)
D_2 - acetylcarnitine	31
D_3 - hexanoylcarnitine	29
D_3 - tetradecanoylcarnitine	26
D_3 - hexadecanoylcarnitine	32
D_8 - valine	41
D_2 - uridine	57

Negative ion mode

ISTD	CV (%)
1,3- ^{15}N - uracil	17
^{13}C - Thymidine	18
5 - bromotryptophan	19
D_4 - glycochenodeoxycholic acid	21
3,3 - dimethylglutaric acid	25
D_4 - tyrosine	27
D_5 - phenylalanine	35

ESTD	CV (%)
D_2 - uridine	29
D_3 - methylmalonic acid	55

Figure S5: Peak area variation of all metabolites identified in the QC sample

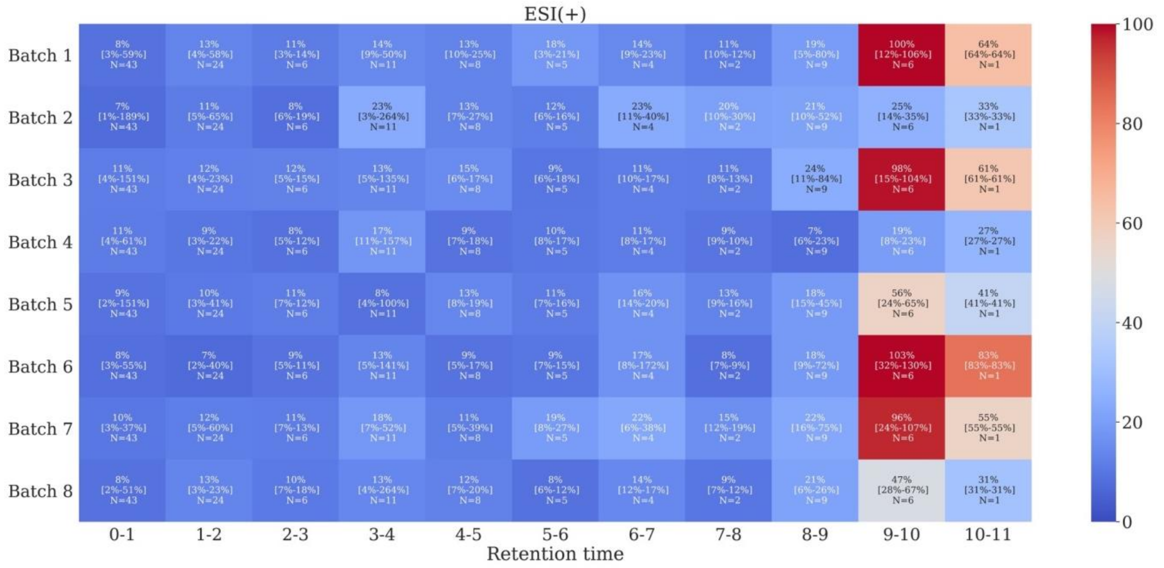

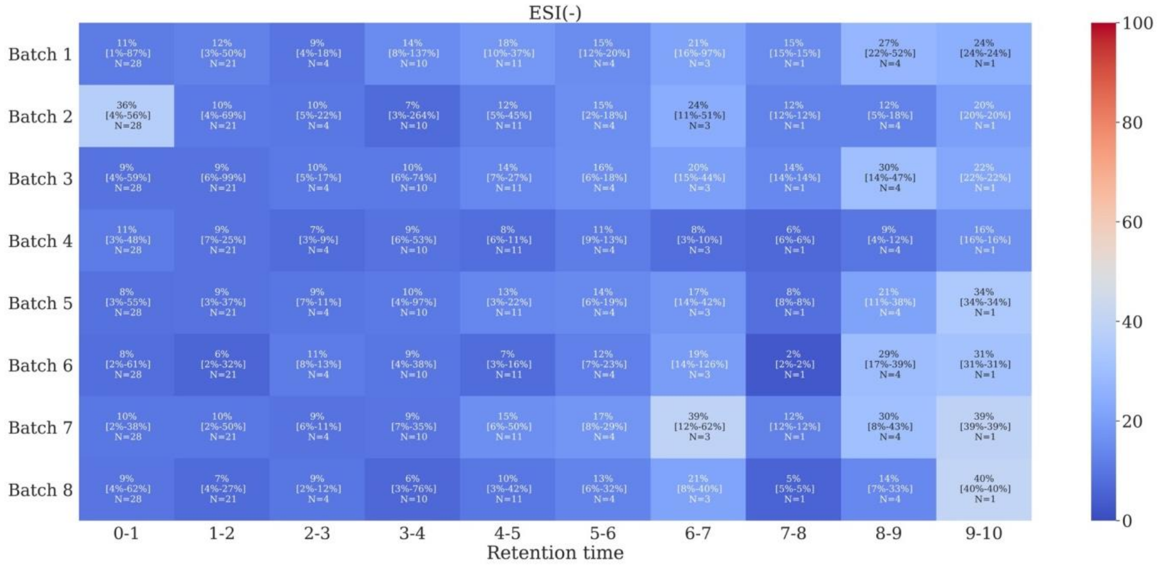

Heat maps showing variation in peak area during a chromatographic run. Panel A: positive ion mode. panel B: negative ion mode. A one-minute bin includes all metabolites eluting within this bin. For every bin the median CV in peak area, range of CV values and number of metabolites is indicated. Colour coding indicates median CV.

Figure S6: Separation of the isomeric pairs isoleucine/leucine and betaine/valine

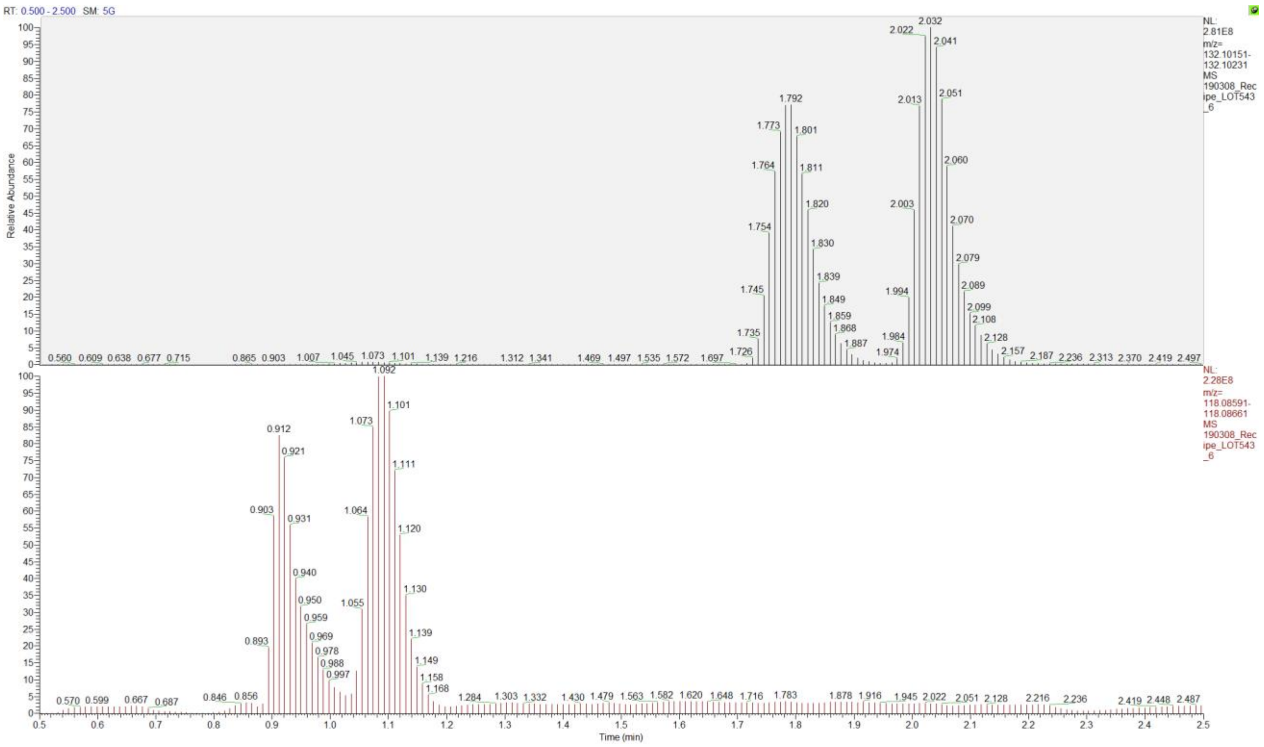

Top panel: XIC of isoleucine (RT: 1.79 min) and leucine (RT: 2.03 min). bottom panel: XIC of betaine (RT: 0.91 min) and valine (RT: 1.09 min)

Table S7: Progenesis QI settings

Data import:	Filter strength: 1
Alignment reference:	Automatic
Peak picking limits:	Automatic sensitivity was set to 3.<br>Minimal peak width: 0.04 minutes
Retention time limits:	Ignore ions before: 0.50 minutes.<br>Ignore ions after: 13.00 minutes
Considered adducts ESI (+):	[M+H], [M+Na], [M+K], [M-e], [M-NH_3+H], [M-H_2O], [M+H+Na], [M+2H], [M+ACN+H], [2M+H], [2M+Na]
Considered adducts ESI (-):	[M-H], [M+Na-2H], [M-H_2O-H], [M-2H], [M-3H], [M+Cl], [M+K- 2H], [M+FA-H], [2M+FA-H]
